# Lidocaine vs. Mometasone Furoate Around the Pediatric Tracheal Tube Cuff: Hemodynamic Stress Response and Postoperative Airway Complications: A Prospective, Randomized, Controlled Study

**DOI:** 10.3390/healthcare13030205

**Published:** 2025-01-21

**Authors:** Ali Ulvi Ölç, Mehmet Yılmaz, Kemal Tolga Saraçoğlu, Ayşe Zeynep Turan Cıvraz, Ayten Saraçoğlu, Paweł Ratajczyk

**Affiliations:** 1 Department of Anesthesiology and Reanimation, Elbistan State Hospital, Kahramanmaras 46300, Turkey; 2 Department of Anesthesiology and Reanimation, Kocaeli City Hospital, Kocaeli 41060, Turkey; drmyilmaz33@gmail.com (M.Y.); ayse.zeynep@gmail.com (A.Z.T.C.); 3 Department of Anaesthesiology, ICU & Perioperative Medicine, Hazm Mebaireek General Hospital HMC, Qatar University College of Medicine, Doha P.O. Box 2713, Qatar; kemaltolgasaracoglu@gmail.com; 4 Department of Anaesthesiology, ICU & Perioperative Medicine, Aisha Bint Hamad Al Attiyah Hospital HMC, Qatar University College of Medicine, Doha P.O. Box 2713, Qatar; saracogluayten@gmail.com; 5 Department of Anesthesiology and Intensive Therapy, Medical University of Lodz, 90-153 Lodz, Poland

**Keywords:** airway, lidocaine, mometasone furoate, intubation, complications, tracheal tube

## Abstract

**Introduction:** According to the results of the APRICOT study, airway and respiratory complications constitute 60% of all anesthesia-related complications and may be life-threatening. The primary aim of this study was to evaluate the effect of lidocaine and mometasone spray on the hemodynamic stress response during tracheal intubation and extubation in children. Our secondary aim was to determine its effect on the incidence of postoperative airway complications. **Materials and Methods:** Following Ethics Committee approval (No: KIIA 2018/489) and clinical trial registration (No: NCT04085744), patient recruitment was initiated only after obtaining parental consent. Children of ASA I-II aged 0 to 16 years and undergoing elective surgery were included. A total of 91 patients were randomly divided into 3 groups. Group M: Patients treated with a topical corticosteroid 0.05% mometasone furoate spray (*n* = 30). Group L: Patients sprayed with 10% lidocaine (*n* = 30). Control group: Patients treated with 0.9% normal saline applied around the cuff (*n* = 31). The systolic, diastolic, and mean blood pressures, heart rate, and SpO_2_ values were recorded before operation, after induction, before and after tracheal intubation, and before and after extubation. Patients were followed up for 24 h postoperatively. **Results:** A statistically significant decrease was found in the lidocaine group for diastolic and mean arterial pressures measured after tracheal intubation (*p* = 0.018 and *p* = 0.027, respectively). There was a significant decrease in heart rate values in Group L after extubation (*p* = 0.024). Cough was observed in 5 patients in the control group at the postoperative 12th hour, but not in the other groups (*p* = 0.009). The distribution of sore throat severity, dyspnea, and hoarseness and the incidence of early postoperative bronchospasm, recorded in all follow-up periods, decreased; however, it did not show a statistically significant difference. **Conclusions:** In conclusion, this study revealed that the topical application of lidocaine and mometasone around the tracheal tube cuff in children not only reduces postoperative cough but also, in the case of lidocaine, suppresses the hemodynamic stress response during both tracheal intubation and extubation.

## 1. Introduction

In recent years, there has been an increase in the incidence of cuffed tube use in pediatric patients. Decreased tracheal tube leakage and lower airway damage are among the reasons for the more frequent use of cuffed tubes [1]. Postoperative airway and respiratory complications may be life-threatening. According to the results of The Anesthesia PRactice In Children Observational Trial (APRICOT) study, airway and respiratory complications constitute 60% of all anesthesia-related complications [2]. Similarly, The Pediatric Difficult Intubation (PeDI) study reports that at least one complication occurs in 20% of difficult intubation cases [3]. The incidence of coughing during emergence from general anesthesia reaches up to 96% in some cases [4]. Postoperative sore throat is a serious complication, with an incidence rate varying from 24% to 44%, and families should be informed about it during the preoperative visit [5,6]. Its occurrence mechanism is explained by direct mucosal inflammation caused by mechanical trauma during tracheal intubation [7]. This event has been more commonly observed in intubated children than in those ventilated with laryngeal masks [8]. A meta-analysis comparing laryngeal mask airway (LMA) to tracheal intubation in pediatric laparoscopic surgeries reported higher odds ratios with tracheal tubes [9].

Studies focusing on preventing postoperative complications are limited in pediatric patients. In some studies, inflating the tracheal tube cuff with lidocaine has been implemented [4,10]. However, insufficient evidence supports its routine use in clinical practice. Nevertheless, the application of lidocaine with endotracheal spray has been widely accepted as a useful method, but it has limitations [11]. Prophylactic use of topical and systemic steroids reduces the incidence of sore throat and cough thanks to the anti-inflammatory effect [12]. Mometasone furoate is a synthetic glucocorticoid that demonstrates a potent anti-inflammatory effect by inhibiting TNF-alpha, interleukin-1, and interleukin-6 when applied topically [13]. Although the topical administration of steroid spray is convenient, cost-effective, and practical in pediatric patients, there is a lack of research on this specific topic in the literature. The primary objective of this study was to evaluate the impact of mometasone furoate (MF) and lidocaine spray on the hemodynamic stress response during tracheal intubation and extubation in children. Our secondary goal was to determine the effect on the incidence of postoperative airway complications.

## 2. Materials and Methods

This research is a prospective, randomized, controlled study. This study was approved by the Kocaeli University School of Medicine Ethics Committee (Date: 30 October 2018 Decision Number: KİA 2018/489). Patient recruitment for the study was initiated after clinical trial registration (No: NCT04085744). Pediatric patients aged 0 to 16 years with American Society of Anesthesiologists (ASA) I-II classification who underwent elective surgery related to ear, nose, and throat, neurosurgery, pediatric surgery, plastic surgery, and orthopedics in the operating rooms of a university hospital between 1 December 2018 and 1 December 2019 and required tracheal intubation were included in the study. Patients undergoing emergency surgery, cases involving the use of nitrous oxide, cases of expected or unexpected difficult intubation, patients undergoing surgeries that required a prone position, patients undergoing oropharyngeal surgery that could cause throat pain due to the surgical field (e.g., tonsillectomy or adenoidectomy), patients undergoing laryngotracheal surgery, and those with allergies to either lidocaine or mometasone were excluded from the study. The study employed high-compliance and low-pressure cuffed tracheal tubes. A total of 91 patients were randomized into three groups, namely Group M, Group L, and the control group. Group M: Patients treated with a topical corticosteroid 0.05% mometasone furoate spray (*n* = 30). Group L: Patients sprayed with 10% lidocaine (*n* = 30). Control group: Patients treated with 0.9% normal saline applied around the cuff (*n* = 31).

Patients and their parents were informed during the preoperative anesthesia visit, and their informed consent was obtained for the study. Randomization was performed using the Random Integer Generator (https:www.random.org, accessed on 25 November 2018) software.

All patients were administered 0.05 mg/kg dormicum intravenously for preoperative premedication and then taken to the operating room. Patients’ demographic data were recorded. All patients were monitored using a 3-lead electrocardiogram, pulse oximeter, and non-invasive blood pressure and temperature checks.

Patients were preoxygenated until the end-tidal oxygen (EtO_2_) value reached 90%. General anesthesia was routinely induced with intravenous administration of 2.5 mg/kg propofol, 1 mcg/kg fentanyl, 0.6 mg/kg rocuronium, and 1 mg/kg lidocaine. Train-of-Four (TOF Watch) monitoring was performed at 1 Hz intervals, followed by tracheal intubation. The tubes were sized for patients using the equation of “size = (age in years/4) + 4”. Tracheal intubations were carried out by pediatric anesthetists using direct laryngoscopy. A size 2 or 3 Macintosh laryngoscope was used for the procedure. The number of intubation attempts was limited to one single attempt. When more than one attempt was taken, it was a case of unexpected difficult intubation, and the patient was excluded from the study. After intubation, the cuff was inflated with air to prevent air leakage from the trachea. The cuff pressure was maintained at 20 to 25 cmH_2_O using a cuff manometer. The surgical procedure was started once the appropriate placement of the tube was confirmed through auscultation, and equal ventilation was ensured to both lungs. Maintenance anesthesia was provided using 2% sevoflurane with a mixture of 40% O_2_ and 60% air in all patients. Routine mechanical ventilator settings were adjusted to a tidal volume of 8 mL/kg and positive end-expiratory pressure (PEEP) of 5 cmH_2_O. Mechanical ventilation was initiated at the conclusion of the operation when the patients were still under deep anesthesia. As spontaneous breathing started, 0.04 mg/kg neostigmine and 0.01 mg/kg atropine were administered intravenously. Patients were extubated once airway reflexes resumed and sufficient tidal volume was achieved, and they were then transferred to the recovery unit with 100% oxygen support. Patients taken to the recovery unit were evaluated with the Modified Aldrete score, which includes 5 parameters including respiration, circulation, activity, consciousness and oxygen saturation. Patients who received 9 points or more from the modified aldrete scoring system were discharged to the ward.

The systolic, diastolic, and mean blood pressures, heart rate, and SpO2 values were documented before the operation, after induction, after tracheal intubation, and before and after extubation. End-tidal CO_2_ was monitored and recorded following intubation. Patients received 10 mg/kg intravenous paracetamol for analgesia. Patients were administered intravenous tramadol at a dose of 1 mg/kg. In this study, we used 10% lidocaine spray (Vemcain VEM İlaç San. ve Tic. A.Ş, Istanbul, Türkiye) and 0.05% mometasone furoate spray (Nazofix Recordati İlaç San. ve Tic. A.Ş, Maslak, Türkiye) for topicalization of tracheal tube cuffs. 

Each puff of the 10% lidocaine spray used in Group L contained 10 mg of lidocaine. A 1.5 mg/kg dose was applied around the cuff before tracheal intubation. In Group M, mometasone furoate was applied as a single puff for children aged 2 to 11 years and two puffs for children aged 11 to 18. Each puff of mometasone furoate contained 50 mcg of the drug substance. In the control group, 0.9% normal saline spray was applied around the cuff.

Patients were followed up through regular ward visits every 6 h for 24 h after surgery, starting from the transfer to the recovery unit. We talked to the patients and their relatives and obtained information from the physicians and nurses in charge of the ward. The following parameters were documented during the follow-up: sore throat, cough, dyspnea, hoarseness, bronchospasm, laryngospasm, aspiration pneumonia, pneumothorax, and need for analgesics.

Sore throat was evaluated with the visual analog scale (VAS) in children 6 years and older. Patients were asked to rate their sore throat on a 10-point VAS scale where 0 = no pain, 1 to 3 = mild pain, 4 to 7 = moderate pain, and 8 to 10 = severe pain. Sore throat in children under 6 years of age was evaluated with the Wong–Baker facial scale [14]. In postoperative follow-ups, constriction, difficulty breathing, increased respiratory effort, involvement of accessory respiratory muscles in breathing, and intercostal retractions were considered dyspnea. For dyspnea treatment, 1 mg/kg intravenous methylprednisolone was administered.

## 3. Statistical Analysis

### Sample Size Calculation

A power analysis was conducted using the G*Power (v3.1.9.2) program to determine the sample size. The power of the study, represented as 1 − β (β = probability of a Type II error), is generally recommended to be 80% for research studies. According to calculations based on Cohen’s effect size coefficients, assuming a large effect size (d = 0.8) for evaluations between two independent groups, it was determined that there needed to be a minimum of 26 participants in each group at a significance level of α = 0.05. Considering the possibility of losses during the study, a decision was made to include 30 participants in each group.

The Number Cruncher Statistical System 2007 (Kaysville, UT, USA) was used for statistical analysis. In the analysis of study data, descriptive statistical methods (mean, standard deviation, median, frequency, ratio, minimum, and maximum) were used. Additionally, for comparing quantitative data, the one-way ANOVA test was employed for three or more groups showing a normal distribution, and the Bonferroni test was used to identify the group causing the difference. The Kruskal–Wallis test was utilized to compare three or more groups not showing a normal distribution, and the Dunn–Bonferroni test was applied to identify the group causing the difference. Qualitative data were compared using Pearson’s Chi-square test and Fisher–Freeman–Halton test. *p* < 0.05 was considered statistically significant.

## 4. Results

**Figure 1:**
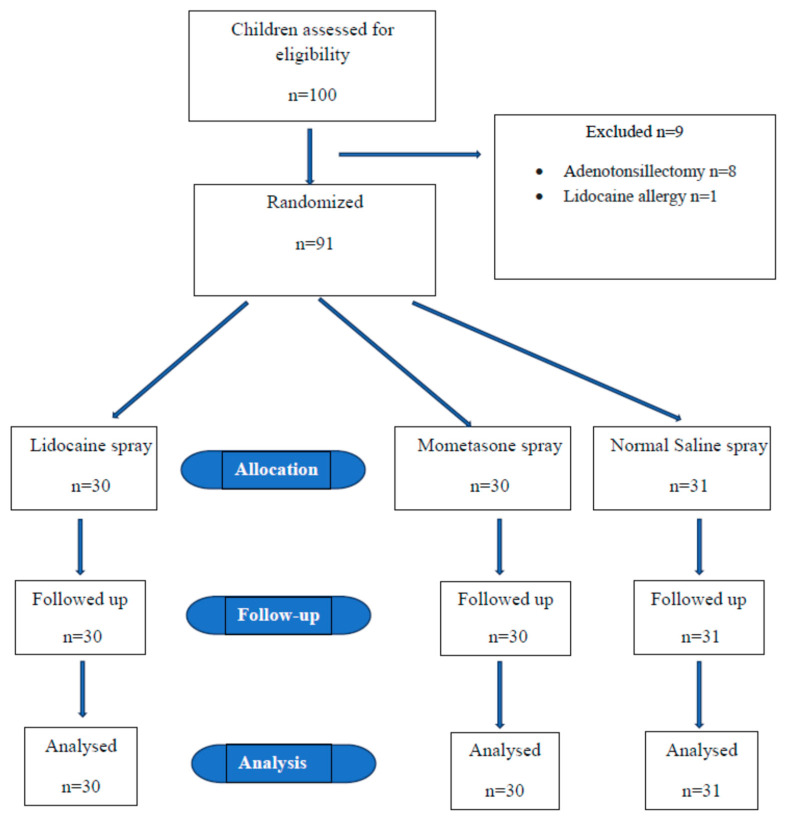
Flow diagram of the participants.

Nine patients were excluded from the study, and the data analysis was ultimately performed on 91 children (Figure 1). Of all patients, 24.2% (*n* = 22) were female and 75.8% (*n* = 69) were male. The ages of the children participating in the study ranged from 3 to 16, with a mean age of 11.6 ± 3.9 years (Table 1). No statistically significant differences were found among the groups in terms of age, gender, body weight, ASA score, duration of surgery, tidal volume on the mechanical ventilator, and distribution of tracheal tube diameter (*p* > 0.05).

There was no statistically significant difference in the hemodynamic values measured during induction and before intubation among the groups (Table 2, *p* > 0.05). While there was no difference in the systolic arterial blood pressure measured after tracheal intubation, statistically significant differences were observed in the lidocaine group’s diastolic and mean arterial pressures (*p* = 0.018 and *p* = 0.027, respectively). According to the results of the pairwise comparisons performed to determine the difference, the post-intubation diastolic blood pressure of patients in Group L was significantly lower than those in the control group and Group M (*p* = 0.027). Similarly, according to the results of pairwise comparisons, the post-intubation OAB value of patients in Group L was significantly lower compared to the patients in Group M and the control group (*p* = 0.041). There was a statistically significant difference in post-extubation peak heart rate measurements among the groups (*p* = 0.022). According to the results of the pairwise comparisons made to determine the difference, the peak heart rate values of Group L patients were significantly lower compared to Group M and the control group (*p* = 0.024). On the other hand, the peak heart rate values measured at all other times did not show a significant difference between the groups (Table 2, *p* > 0.05).

There was no statistically significant difference among the groups regarding peripheral oxygen saturation and end-tidal CO_2_ levels measured at all times (Table 3, *p* > 0.05). The groups did not show a significant difference in the incidence of coughing during emergence and at the sixth postoperative hour (Table 4, *p* > 0.05). Statistically significant differences were observed among the groups regarding the incidence of cough at the 12th hour after the operation (*p* = 0.009, *p* < 0.01). Five patients in the control group developed coughing at the 12th postoperative hour, while no coughing was observed in the other groups (*p* = 0.009). There was no statistically significant difference between the groups in the distributions of sore throat pain, dyspnea, hoarseness, and the incidence of early postoperative bronchospasm recorded in all follow-up periods (*p* > 0.05).

## 5. Discussion

In this prospective randomized controlled study, lidocaine or MF spray was administered around the tracheal tube cuff before tracheal intubation in pediatric patients undergoing elective surgery under general anesthesia. Patients were compared with subjects in the control group for perioperative hemodynamic variables and airway complications developing in the first 24 h after the operation. According to the results of our study, lidocaine and MF significantly reduced the incidence of cough at the postoperative 12th hour. No significant difference was noted in the incidence of sore throat, dyspnea, hoarseness, bronchospasm, laryngospasm, aspiration pneumonia, or pneumothorax. There was no increase in the diastolic and mean arterial blood pressure values measured immediately after intubation in the lidocaine group. Also, the heartbeat rates remained lower after extubation.

As it is during tracheal intubation, one of the primary challenges encountered during extubation is the stimulation of laryngeal reflexes and sympathetic discharge [15]. Local anesthetics, beta-blockers, calcium channel blockers, or opioids have been used to suppress the hemodynamic changes occurring during tracheal intubation and extubation [16]. However, the side effects of systemic agents will generally be greater than those of topically applied agents. Intracuff alkalinized lidocaine has been used in some studies to reduce postoperative complications [17]. However, the results of the studies on this topic are controversial. Ahmady MS et al. [18] demonstrated a reduction in the incidence of cough during extubation and in the post-anesthesia care unit in children when the cuffs were inflated with lidocaine. However, there was a significant prolongation in the onset of spontaneous ventilation before extubation compared to the control group. During intracuff local anesthetic applications, there is always a risk of endotracheal cuff rupture and overdose. Another limitation of intracuff lidocaine application is the time required for lidocaine to diffuse through the cuff membrane. Another study involving children intubated with lidocaine-filled cuffed tracheal tubes for adenotonsillectomy reported a higher coughing incidence than the normal saline group [19]. The reason was associated with the short duration of surgery.

The effect of agents topically sprayed around the cuff starts immediately. For this reason, such agents can be effectively used in short-duration surgeries. Our study did not include children undergoing adenotonsillectomy to avoid the impact on postoperative follow-up for sore throat. The shortest duration of surgery was 45 min, and even in these patients, the incidence of postoperative cough was successfully reduced.

Soares SMF et al. [10] reported that when the cuff was inflated with lidocaine, the hemodynamic response to tracheal extubation could be suppressed compared to inflation with saline or air, and the incidence of sore throat was lower in the early postoperative period. However, the use of nitrous oxide in this study was up to the choice of the anesthetists. Therefore, the rapid diffusion of nitrous oxide into the cuff might have compromised standardization in patients in that study. In our study, however, we opted not to use nitrous oxide to prevent any increase in intracuff pressure, reinforcing the reliability of our results. The authors also excluded the surgeries that lasted longer than 60 min from this study. This decision was made because lidocaine administered into the cuff requires more than 60 min for diffusion to reach the tracheal mucosa.

In Bayesian network meta-analysis, inhaled corticosteroids have been reported as the most effective topical agent in reducing postoperative sore throat in the early postoperative period [20]. Both lidocaine and corticosteroids have analgesic and anti-inflammatory properties. Mometasone spray is a corticosteroid that inhibits the influx of inflammatory cells into the mucosa and has been proven to reduce postoperative sore throats in adults [21]. To the best of our knowledge, the present study is the first to investigate the effectiveness of mometasone spray in preventing airway complications in pediatric patients. Mometasone reduced sore throat as effectively as topical lidocaine. Similarly, it has been found to be non-inferior to lidocaine in preventing postoperative hoarseness and bronchospasm [21]. We believe lidocaine’s superiority in providing hemodynamic stability during intubation and extubation is due to its topical anesthetic effect on the laryngotracheal mucosa, allowing for better tolerance to the tracheal tube.

In many studies, topical lidocaine has been administered in the form of a spray onto the vocal cords in the trachea. However, the anesthesia of the airway with local anesthetic spray can lead to laryngospasm [22]. Laryngospasm is a serious and potentially fatal complication that can lead to partial or complete airway obstruction. In our study, topical agents were applied around the tracheal tube cuff, not directly to the vocal cords or larynx, in order to prevent laryngospasm. As a result, none of our patients developed laryngospasm. Bronchospasm was observed in one patient from each group, but it was successfully managed with symptomatic treatment.

In a meta-analysis investigating the efficacy of topical corticosteroids on postoperative sore throat with 20 studies including a total of 2200 patients, betamethasone was used in 13 studies, dexamethasone in 4 studies, triamcinolone in 2 studies, and beclomethasone in 1 study [23]. Corticosteroids applied to the tracheal tube have been found to be more effective than topical lidocaine. The efficacy of topical corticosteroids is directly proportional to their lipophilicity. Preparations with high lipophilic properties have increased absorption and receptor affinity. They bind strongly to plasma proteins, resulting in less systemic circulation. The furoate ester side chain in MF’s structure imparts a high degree of lipophilicity. Among topical corticosteroids, MF has the highest lipophilicity [24,25]. However, a significant advantage of MF over other topical corticosteroids is that it is more reliable in children. Its highly lipophilic nature and minimal systemic absorption in children make it a viable option. Its other unique advantages are rapid hepatic biotransformation and the lack of impact on the hypothalamic–pituitary–adrenal axis [26].

## 6. Limitations

The most significant limitations of this study are its single-center design, the fact that it is not a double-blind trial, and the relatively low sample size. Further research is needed to investigate the reliability of both lidocaine and MF in children.

## 7. Conclusions

In conclusion, it has been demonstrated that the topical application of lidocaine and MF around the tracheal tube cuff reduces postoperative airway complications, particularly cough, in children. Moreover, lidocaine has demonstrated the additional benefit of suppressing the hemodynamic stress response during tracheal intubation and extubation.

## Figures and Tables

**Table 1:** Demographic data, duration of surgery, mean tidal volume and tracheal tube diameter of the patients in the groups.

			Group		
		Total	Mometasone	Lidocaine	Control
Age ^‡^ (years)		11.6 ± 3.9	11.1 ± 3.9	11.8 ± 3.6	11.8 ± 4.1
Gender ^§^	Female	22 (24.2)	5 (16.7)	7 (23.3)	10 (32.3)
	Male	69 (75.8)	25 (83.3)	23 (76.7)	21 (67.7)
ASA Class ^§^	I	77 (84.6)	25 (83.3)	27 (90.0)	25 (80.6)
	II	14 (15.4)	5 (16.7)	3 (10.0)	6 (19.4)
Weight ^‡^ (kg)		41.9 ± 14.5	41.9 ± 16.2	45.3 ± 14.9	38.6 ± 12.0
Duration of the operation ^‡^ (min.)		93.8 ± 46.9	84.2 ± 40.5	88.4 ± 34.2	108.4 ± 59.5
Tidal Volume ^‡^ (mL)		329.1 ± 105.5	326.5 ± 117.3	353 ± 104.5	308.5 ± 92.3
Tube diameter ^§^ (mm)	3.5	3 (3.3)	2 (6.7)	0 (0)	1 (3.2)
	4	2 (2.2)	0 (0)	1 (3.3)	1 (3.2)
	4.5	13 (14.3)	4 (13.3)	4 (13.3)	5 (16.1)
	5	9 (9.9)	1 (3.3)	5 (16.7)	3 (9.7)
	5.5	12 (13.2)	7 (23.3)	2 (6.7)	3 (9.7)
	6	15 (16.5)	4 (13.3)	6 (20)	5 (16.1)

ASA: American Society of Anesthesiologists data are presented as *n* (%) proportions ^§^, mean (SD) ^‡^.

**Table 2:** Comparison of hemodynamic values of the groups.

		Group				Test Value
		Total (*n* = 91) Mean ± SD	Corticosteroid (*n* = 30) Mean ± SD	Lidocaine (*n* = 30) Mean ± SD	Control (*n* = 31) Mean ± SD	*p*
Systolic Blood Pressure (mmHg)	Preoperative	123.0 ± 13.2	126.2 ± 12.4	121.4 ± 14.1	121.4 ± 12.7	^a^ 0.268
	After Induction	108.9 ± 13.3	108.9 ± 12.61	108.9 ± 14.3	108.8 ± 13.4	^a^ 0.999
	After Intubation	122.9 ± 14.9	125.3 ± 14.6	119.3 ± 14.9	124.1 ± 15.1	^a^ 0.258
	Before Extubation	109.1 ± 14.2	109.9 ± 15.0	110.5 ± 16.1	107.1 ± 11.1	^a^ 0.602
	After Extubation	123.1 ± 14.4	123.2 ± 14.6	121.2 ± 14.4	125.0 ± 14.6	^a^ 0.592
Diastolic Blood Pressure (mmHg)	Preoperative	75.4 ± 10.6	77.5 ± 12.6	74.0 ± 9.43	74.6 ± 9.7	^a^ 0.413
	After Induction	63 ± 12.7	64.6 ± 12.9	62.1 ± 10.2	62.2 ± 14.7	^a^ 0.697
	After Intubation	776.6 ± 15.9	79.2 ± 15.2	70.0 ± 14.3	80.6 ± 16.5	^a^ 0.018*
	Before Extubation	65.0 ± 12.1	67.9 ± 13.8	64.0 ± 11.9	63.1 ± 10.4	^a^ 0.272
	After Extubation	75.6 ± 13.9	76.5 ± 17.6	72.6 ± 12.7	77.5 ± 10.8	^a^ 0.363
Mean Arterial Pressure (mmHg)	Preoperative	93.3 ± 11.4	96.3 ± 12.5	90.6 ± 10.3	93.1 ± 10.8	^a^ 0.154
	After Induction	80.2 ± 12.1	81.4 ± 12.5	78.4 ± 10.7	80.7 ± 13.4	^a^ 0.617
	After Intubation	95.1 ± 16.0	98.9 ± 16.9	88.8 ± 14.9	97.6 ± 14.8	^a^ 0.027 *
	Before Extubation	81.8 ± 12.4	84.3 ± 13.6	81.5 ± 13.9	79.7 ± 9.2	^a^ 0.348
	After Extubation	94.6 ± 12.8	96.8 ± 13.4	92.3 ± 12.2	94.7 ± 12.9	^a^ 0.397
Heart Rate (beat/min)	Preoperative	103.3 ± 18.3	105.2 ± 21.4	1103.3 ± 18.4	101.5 ± 15.1	^a^ 0.744
	After Induction	99.9 ± 16.8	103.1 ± 19.5	97.9 ± 13.0	98.7 ± 17.3	^a^ 0.431
	After Intubation	110.2 ± 16.8	114.1 ± 19.2	104.9 ± 14.8	111.6 ± 15.2	^a^ 0.088
	Before Extubation	91.4 ± 19.9	95.5 ± 22.8	88.7 ± 17.9	90.2 ± 18.6	^a^ 0.384
	After Extubation	105.0 ± 17.5	112 ± 16.5	100.1 ± 18.8	103.0 ± 15.4	^a^ 0.022 *

^a^ One-way ANOVA,* *p* < 0.05.

**Table 3:** SpO_2_ and EtCO_2_ variations among the groups during the induction of general anesthesia, tracheal intubation, and extubation.

			Group			Test Value
		Total (*n* = 91) Mean ± SD	Corticosteroid (*n* = 30) Mean ± SD	Lidocaine (*n* = 30) Mean ± SD	Control (*n* = 31) Mean ± SD	*p*
Peripheral Oxygen Saturation (%)	Preoperative	99.2 ± 1.3	99.4 ± 0.8	99.1 ± 1.9	99.0 ± 0.9	^d^ 0.055
	After Induction	99.8 ± 0.42	99.9 ± 0.2	99.9 ± 0.2	99.7 ± 0.6	^d^ 0.346
	After Intubation	99.8 ± 0.5	99.9 ± 0.7	99.8 ± 0.3	99.8 ± 0.4	^d^ 0.612
	Before Extubation	99.8 ± 0.4	100 ± 0	99.8 ± 0.5	99.7 ± 0.5	^d^ 0.052 *
	After Extubation	99.7 ± 0.7	99.9 ± 0.2	99.8 ± 0.3	99.5 ± 1.1	^d^ 0.406
EtCO_2_ (mm Hg)	After Induction	31.9 ± 4.5	31.4 ± 4.7	32.2 ± 4.5	32.0 ± 4.5	^a^ 0.758
	After Intubation	36.3 ± 4.0	37.3 ± 4.5	35.5 ± 4.4	36.3 ± 3.0	^a^ 0.233
	Before Extubation	32.2 ± 3.6	32.6 ± 3.9	31.8 ± 3.4	32.42 ± 3.6	^a^ 0.662
	After Extubation	34.7 ± 4.7	35.9 ± 4.7	33.8 ± 4.7	34.6 ± 4.5	^a^ 0.224

^d^ Kruskal–Wallis test, ^a^ one-way ANOVA * *p* < 0.05.

**Table 4:** Comparison of postoperative complications among the groups at various follow-up intervals.

Complication	Follow-Up Time Point		Group				
			Total	Corticosteroid	Lidocaine	Control	*p*
			(*n* = 91) %	(*n* = 30) %	(*n* = 30) %	(*n* = 31) %	
Cough (patients, *n*)	Recovery Unit		21 (23.1)	4 (13.3)	11 (36.7)	6 (19.4)	^b^ 0.083
	Hour 6		18 (19.8)	5 (16.7)	3 (10.0)	10 (32.3)	^b^ 0.081
	Hour 12		5 (5.5)	0 (0.0)	0 (0.0)	5 (16.1)	^c^ 0.009 *
Sore Throat Pain (patients, *n*)	Recovery Unit	No pain	67 (73.6)	24 (80.0)	19 (63.3)	24 (77.4)	^c^ 0.306
		Mild Pain	17 (18.7)	3 (10.0)	9 (30.0)	5 (16.1)	
		Moderate Pain	6 (6.6)	3 (10.0)	2 (6.7)	1 (3.2)	
		Severe Pain	1 (1.1)	0 (0.0)	0 (0.0)	1 (3.2)	
	Hour 6	No pain	72 (79.1)	21 (70.0)	27 (90.0)	24 (77.4)	^c^ 0.101
		Mild Pain	17 (18.7)	9 (30.0)	3 (10.0)	5 (16.1)	
		Moderate Pain	2 (2.2)	0 (0.0)	0 (0.0)	2 (6.5)	
		Severe Pain	0 (0.0)	0 (0.0)	0 (0.0)	0 (0.0)	
	Hour 12	No pain	87 (95.6)	30 (100.0)	29 (96.7)	28 (90.3)	^c^ 0.323
		Mild Pain	4 (4.4)	0 (0.0)	1 (3.3)	3 (9.7)	
		Moderate Pain	0 (0.0)	0 (0.0)	0 (0.0)	0 (0.0)	
		Severe Pain	0 (0.0)	0 (0.0)	0 (0.0)	0 (0.0)	
	Hour 24	No Pain	90 (98.9)	30 (100.0)	30 (100.0)	30 (96.8)	^c^ 1.000
		Mild Pain	1 (1.1)	0 (0.0)	0 (0.0)	1 (3.2)	
		Moderate Pain	0 (0.0)	0 (0.0)	0 (0.0)	0 (0.0)	
		Severe Pain	0 (0.0)	0 (0.0)	0 (0.0)	0 (0.0)	
Dyspnea (patients, *n*)	Recovery Unit		1 (1.1)	0 (0.0)	1 (3.3)	0 (0.0)	^c^ 0.664
Hoarseness (patients, *n*)	Recovery Unit		12 (13.2)	3 (10.0)	4 (13.3)	5 (16.1)	^c^ 0.927
	Hour 6		3 (3.3)	1 (3.3)	0 (0.0)	2 (6.5)	^c^ 0.773
Bronchospasm (patients, *n*)	Recovery Unit		3 (3.3)	1 (3.3)	1 (3.3)	1 (3.2)	^c^ 1.000

Units are number of patients. ^b^ Pearson’s Chi-Square test, ^c^ Fisher–Freeman–Halton test * *p* < 0.01.

## Data Availability

Data are contained within the article.
